# ScMicrobesAtlas: A comprehensive microbial atlas at single-cell resolution in human disease contexts

**DOI:** 10.1016/j.gendis.2025.101594

**Published:** 2025-03-08

**Authors:** Jinyang Liu, Rui Hou, Lei Ji, Yue Gong, Pan Li, Peiwen Liu, Jinxin Dong, Hongzhe Guo, Yun Zhang, Junlin Xu, Tingting Hui, Haotian Tian, Yankun Liu, Meijun Zhang, Geng Tian, Jialiang Yang

**Affiliations:** a Geneis Beijing Co., Ltd., Beijing 100102, China; b College of Computer Science and Electronic Engineering, Hunan University, Changsha, Hunan 410082, China; c Beijing Zhilan Technology Co., Ltd., Beijing 102206, China; d Department of Medical Molecular Diagnosis, Tangshan People's Hospital, Tangshan, Hebei 063001, China; e Tangshan Key Laboratory of Precision Medicine Testing, Tangshan People's Hospital, Tangshan, Hebei 063001, China

The microbiome plays a significant role in human health and disease.^1^ Although bulk tissue analyses have identified disease-specific microbial signatures, these studies do not capture microbial-host cell enrichments and their associations with cell-type-specific activities.^2^, ^3^, ^4^ Thus, we present the Single Cell Microbes Atlas (ScMicrobesAtlas, http://scmbdb.geneis.org.cn:8089), a comprehensive microbial atlas that provides single-cell resolution insights into various human diseases. To date, ScMicrobesAtlas version 1.0 integrates 318 samples from 32 single-cell RNA sequencing datasets, encompassing 21 disease types, and uncovers interactions between 611 bacterial genera and over 1.3 million human cells. All the data were uniformly processed with a standardized workflow, including quality control, batch effect removal, clustering, cell-type annotation, microbial signal identification and quantification, differential expression analysis, and functional enrichment analysis (Fig. 1A). ScMicrobesAtlas facilitates the comparative analysis of microbiome composition and identifies both shared and cell-type-specific microbial enrichments across diverse cell types and disease states. Additionally, it allows users to assess gene expression alterations and pathway activities in specific cell types in response to a particular microbiome. In summary, ScMicrobesAtlas offers a valuable resource for researchers to investigate disease-microbiome associations at the single-cell resolution.

**Figure 1:**
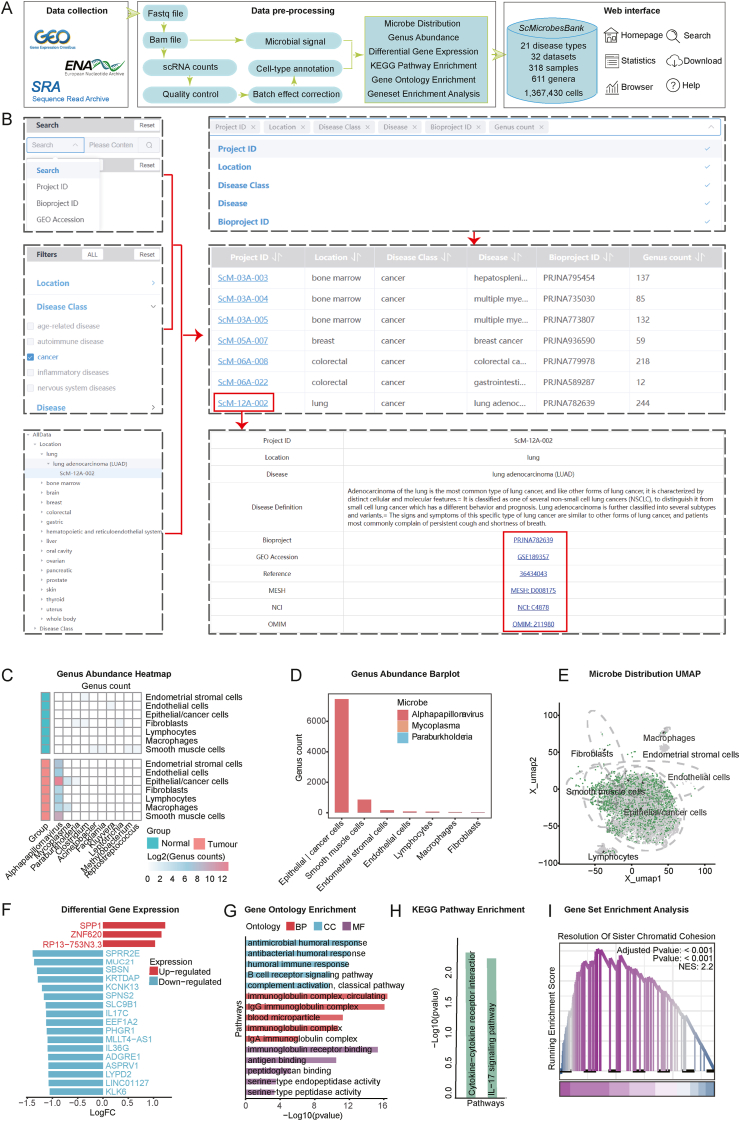
ScMicrobesAtlas provides a valuable resource for researchers to investigate disease-microbiome associations at the single-cell resolution. **(A)** Schematic representation of ScMicrobesAtlas, encompassing data collection, preprocessing, and web interface. **(B)** Illustration of sample selection and filtering functions. **(C–I)** Seven interactive modules within ScMicrobesAtlas facilitate comprehensive and in-depth exploration of disease-microbiome associations: (C) Genus Abundance Heatmap module; (D) Genus Abundance Barplot module; (E) Microbe Distribution UMAP module; (F) Differential Gene Expression module; (G) Gene Ontology Enrichment module; (H) KEGG Pathway Enrichment module; and (I) Gene Set Enrichment Analysis module.

ScMicrobesAtlas is a comprehensive database that seeks to serve as a one-stop resource for researchers investigating the associations between disease and the microbiome at single-cell resolution. Its interactive web interface offers a range of advanced visualization tools.

The search page allows users to efficiently retrieve data using flexible “search” and “filter” functions (Fig. 1B). Users can search for specific tissue types and diseases. The search results display key information for each project, including the disease class, disease name, genus count, and a “Project ID” hyperlink to access detailed results of the corresponding project, which includes the disease description, literature reference, and cell and sample information. The tree browser organizes the extensive database content in an intuitive tree structure. Clicking on a disease class expands the tree to show specific diseases within that class.

The ScMicrobesAtlas provides users with seven interactive modules to facilitate the comprehensive and in-depth exploration of disease-microbiome associations. To demonstrate the utility and potential applications of these modules, we used the ScM-19A-017 project as an example for studying disease-microbiome associations at single-cell resolution.

In the “Genus Abundance Heatmap” module**,** the raw infection counts of each microbial genus across the various cell types can be visualized using a heatmap. This heatmap representation allows users to compare the microbial composition between the original research sample groups or across cell types, such as healthy versus disease samples and tumor versus adjacent normal tissues. Through this analysis, users can discover microbial taxa that are shared across cell types as well as those that are enriched in a cell-type-specific manner under different disease states. For example, we identified a higher abundance of microbial populations in cervical cancer tumor tissues and their near absence in adjacent normal samples (Fig. 1C).

In the “Genus Abundance Barplot” module, stacked bar plots depict the raw infection counts for each cell type. These visualizations highlight the distribution of microbes across the different host cell types within the disease states. By comparing the microbial composition across cell types, users can identify the specific cell populations that are preferentially infected by particular microbes. For instance, epithelial/cancer cells and smooth muscle cells exhibited greater microbial abundance and stronger enrichment of certain taxa compared with other cell types. Notably, alpha-papillomavirus, a primary causative agent of cervical cancer, was found to dominate the microbial composition in epithelial/cancer cells and smooth muscle cells (Fig. 1D).

In the “Microbe Distribution UMAP” module**,** the UMAP plot shows the identities and clustering of cells based on their transcriptomic profiles. Users can select specific microbes from a drop-down menu to visualize the infection status of those microbes across all cells, revealing infected and uninfected cell populations in the UMAP plot (Fig. 1E).

In the “Differential Gene Expression” module**,** the bar plot displays the differential expression genes in a particular cell type between specific microbe-infected and uninfected cells. This visualization allows users to quickly identify the transcriptional changes occurring in host cells in response to specific microbial infections. This can provide valuable insights into the host-microbiome interactions within the disease context and shed light on their potential roles in disease pathogenesis. Using this module, we found that SPP1 expression was up-regulated in epithelial/cancer cells after alpha-papillomavirus infection in tumors (Fig. 1F). This finding is consistent with the report of recent literature that macrophages and epithelial cells interact through the SPP1-CD44 axis, which might contribute to the cervical carcinogenesis.^5^

In the “Gene Ontology Enrichment” module, the bar plot displays the significantly enriched gene ontology pathways based on the cell-type- and microbe-specific differential expression genes (Fig. 1G). This analysis allows users to identify the specific biological processes and molecular functions that are perturbed in particular cell types in response to microbial infection. For example, in epithelial/cancer cells from tumors infected with alpha-papillomavirus, these differential expression genes were significantly enriched in the gene ontology pathways of antimicrobial humoral response, humoral immune response, immunoglobulin complex, and immunoglobulin receptor binding (Fig. 1G).

In the “KEGG Pathway Enrichment” module, the bar plot displays the significantly enriched Kyoto Encyclopedia of Genes and Genomes (KEGG) pathways using the cell-type- and microbe-specific significantly differential expression genes. This complementary analysis enables the identification of dysregulated biological pathways and signaling cascades in particular cell types upon microbial infection. For example, the KEGG enrichment analysis demonstrated that in epithelial/cancer cells from tumors infected with alpha-papillomavirus, these differential expression genes were significantly enriched in immune-related pathways associated with the development and progression of cervical cancer, such as cytokine–cytokine receptor interaction and IL-17 signaling (Fig. 1H).

In the “Gene Set Enrichment Analysis” module, the GSEA plot highlights the activation of particular biological pathways in a select cell type upon specific microbial infection. This analysis allows users to identify cell-type-specific disease-related biological processes that are perturbed in response to microbial infection. For example, in epithelial/cancer cells from tumors infected with alpha-papillomavirus, there was increased activity of multiple hallmarks of tumor pathways related to cell cycle progression and DNA damage and repair. Specifically, the activated pathways included E2f targets, G2m checkpoint, and diseases of DNA repair (Fig. 1I).

## CRediT authorship contribution statement

**Jinyang Liu:** Writing – review & editing, Writing – original draft, Methodology, Investigation. **Rui Hou:** Supervision, Investigation, Data curation. **Lei Ji:** Visualization, Methodology. **Yue Gong:** Visualization, Formal analysis. **Pan Li:** Visualization, Formal analysis. **Peiwen Liu:** Visualization, Validation. **Jinxin Dong:** Visualization, Validation. **Hongzhe Guo:** Visualization, Validation. **Yun Zhang:** Visualization, Validation. **Junlin Xu:** Funding acquisition. **Tingting Hui:** Visualization, Validation. **Haotian Tian:** Software. **Yankun Liu:** Resources, Visualization, Writing – review & editing. **Meijun Zhang:** Visualization, Validation. **Geng Tian:** Visualization, Validation. **Jialiang Yang:** Resources, Conceptualization.

## Funding

This work was supported by the 10.13039/501100001809National Natural Science Foundation of China (No. 62302156), the Natural Science Foundation of Hunan Province, China (No. 2023JJ40180), and Hebei Province Talent Introduction Project (No. 25120201B) “Identification of Key Genes in CAFs of Colorectal Cancer Liver Metastasis Using Single-Cell Sequencing Technology” (China).

## Conflict of interests

The authors declared no conflict of interests.

## References

[1] Young V.B. (2017). The role of the microbiome in human health and disease: an introduction for clinicians. Br Med J.

[2] Narunsky-Haziza L., Sepich-Poore G.D., Livyatan I. (2022). Pan-cancer analyses reveal cancer-type-specific fungal ecologies and bacteriome interactions. Cell.

[3] Ghaddar B., Biswas A., Harris C. (2022). Tumor microbiome links cellular programs and immunity in pancreatic cancer. Cancer Cell.

[4] Niño J.L.G., Wu H., LaCourse K.D. (2022). Effect of the intratumoral microbiota on spatial and cellular heterogeneity in cancer. Nature.

[5] Sheng B., Pan S., Ye M. (2024). Single-cell RNA sequencing of cervical exfoliated cells reveals potential biomarkers and cellular pathogenesis in cervical carcinogenesis. Cell Death Dis.

