# Supplementary material for: ScMicrobesAtlas: A comprehensive microbial atlas at single-cell resolution in human disease contexts
Source: Genes Dis. 2025 Mar 8;13(1):101594. doi: 10.1016/j.gendis.2025.101594 (PMC12466128; doi:10.1016/j.gendis.2025.101594)
Supplement: Multimedia component 1 [file mmc1.docx]

**ScMicrobesAtlas: a comprehensive microbial atlas at single-cell resolution in human disease contexts**

**Materials and methods**

**ScRNA-seq data collection**

We systematically collected scRNA-seq data by searching PubMed for the literature related to scRNA-seq research using ((cancer or disease[Title/Abstract]) AND (patient)) AND (single cell RNA sequencing) as keywords. To achieve a comprehensive characterization of the TME, this study excluded any studies involving cell sorting operations prior to sequencing by manually reviewing the method section of the literature. The remaining literature was then manually confirmed if the raw FASTQ data generated by the CellRanger mkfastq command were publicly available from the sequence read archive (SRA) and European Nucleotide Archive (ENA) repository. We downloaded the data from SRA and ENA, and manually mapped FASTQ files to the corresponding samples. By manually reviewing each literature and supplemental materials, we collected the clinical information of patients and samples in each dataset, including age, gender, and tissue type. In this study, we followed the sample grouping based on the original article, and no exclusion or randomization of samples was required. Additionally, we did not perform a power analysis on the sample size as it was deemed irrelevant to the objectives of our study and the status of the samples were not blind to the analysis.

**Microbial data extraction**

The pipeline for extracting microbial data from scRNA-seq data involves several steps, as described by the Single-cell Analysis of Host-Microbiome Interactions (SAHMI) method. The input data consists of the FASTQ files collected by the above procedure. Then, BAM files are generated using the CellRanger count command. The PathSeq tool (https://software.broadinstitute.org/pathseq/) is used to identify microbial sequences in the possorted_genome_bam.bam file. Based on the cell barcode and unique molecular identifiers (UMI) in each paired sequence, the abundance and cellular source of every microbial sequence are inferred.

**Data quality control and batch effect correction**

The expression matrix of scRNA-seq could be downloaded from the Gene Expression Omnibus (GEO), the website provided by the original paper, or the output of the CllRanger count command. Using the quality control parameters and scanpy (v1.8.2), we filtered low-quality cells and rarely expressed genes. For each dataset, after normalisation to UPM (UMI per million) and integrated across samples, the batch effect removal was performed using Harmony and BBKNN.

**Cell clustering and annotation**

BBKNN constructs a k-nearest-neighbour graph for the whole dataset considering the batch effect. Using this graph, scanpy can perform Leiden, an unsupervised graph-based clustering method, to cluster cells. The cell type annotation of scRNA-seq data is processed following the methods described in the corresponding paper. Sometimes the metadata provided by the original authors already contain the cell type annotations and we prefer to use them instead of re-doing the whole procedure.

**Visualization of individual cells**

Cell-type visualization of a dataset was performed using both t-distributed stochastic neighbour embedding (t-SNE) and the uniform manifold approximation and projection (UMAP) method. UMAP was generated by the graph from BBKNN.

**NEBULA analysis of scRNA-seq data**

The negative binomial mixed model using large sample approximation (NEBULA) is a new fast algorithm for differential gene expression analysis of scRNA-seq data. By combining the microbial and scRNA-seq data, we identified infected and uninfected cells of the same cell type for a microorganism genus. In cases where the cell counts of infected or uninfected cells within a cell type exceeded 3, we proceeded with the differential expression analysis. The Benjamini-Hochberg method was employed to adjust the combined p-values, and genes with |log(Fold change)| ≥ 1 and adjusted P < 0.05 were considered statistically significant.

**Differential expression genes (DEGs) pathways enrichment analysis**

Gene Ontology (GO) and Kyoto Encyclopedia of Genes and Genomes (KEGG) pathways enrichment analysis was carried out to seek related biological pathways using the R package "clusterProfiler". GO Enrichment included biological process (BP), cellular component (CC) and molecular function (MF). P<0.05 were recognized as significant.

**Gene set enrichment analysis (GSEA)**

GSEA was conducted using the R package "clusterProfiler" to investigate enriched pathways. The analysis utilized REACTOME, KEGG, PID and hallmark gene sets from the Molecular Signatures Database (v.7.1). The genes were ranked based on their logFC values to assess their enrichment in specific pathways.

**Database construction**

ScMicrobesAtlas is freely available at http://scmbdb.geneis.org.cn:8089. The front-end of ScMicrobesAtlas website was developed using Vue 3.3.0 and Element Plus 2.3.5. The back-end of the website was developed using java and springboot. Data storage and management were performed using MySQL v5.7. ECharts v5.4.2 plugin software was used to create interactive tables and results visualization. All upstream and downstream analyses were performed using R 4.1.2 and Python 2.7 based on the Linux system. All data in ScMicrobesAtlas is available to the users in the ‘Download’ page. ScMicrobesAtlas website can be visited on popular web browsers, such as Google Chrome, Firefox, Microsoft Edge, and Safari.

**Comparison across datasets**

ScMicrobesAtlas offers a utility for cross-dataset comparison, enabling users to analyze differences in microbial composition, gene expression alterations, and pathway activities among samples across various cancer types or tissues. We utilize Staphylococcus as a case study to illustrate the utility and potential application.

Staphylococcus was a prevalent genus of cocci commonly found on human skin and in the nasal cavity. It was closely associated with a range of diseases and can initiate systemic inflammatory responses. Furthermore, research suggests that Staphylococcus infections may be linked to the development of specific cancers, particularly colorectal^1-3^ and gastric cancers (GC)^4-6^. Through our search of the ScMicrobesAtlas database, we identified a higher abundance of Staphylococcus in patients with systemic lupus erythematosus (SLE) (ScM-06A-008), GC with lymph node metastasis (ScM-08A-012), as well as in right-sided colorectal cancer (CRC).

In SLE patients, Staphylococcus was predominantly detected in LEG, monocytes, B cells, and T cells (Figure S1A). Notably, the expression of MICB was upregulated in Staphylococcus-infected B cells compared to uninfected controls (Figure S1B), corroborating previous findings that link MICB to SLE^7,8^. KEGG and GO enrichment analyses revealed disruptions in the peroxisome and bile acid secretion pathways following Staphylococcus infection (Figure S1C and S1D). Peroxisomes are essential organelles involved in redox signaling and lipid homeostasis, and research has associated alterations in the redox state and oxidative stress with SLE^9-11^. Additionally, GESA demonstrated activate of microbiome-related inflammation and metabolism pathways, such as interleukin-17 signaling and tyrosine metabolism (Figure S1E). These findings were consistent with previous reports that Staphylococcus promoted SLE-like autoimmune inflammation through the IL-23/IL-17 axis^12^. Additionally, there were evidence of immunometabolic dysregulation of CD11c+ T-bet+ B cells in patients with SLE^13^. Clinically, Bruton's Tyrosine Kinase (BTK) inhibitors have been approved for the treatment of autoimmune diseases, and patients can benefit from these therapies^14,15^.

In GC patients with lymph node metastasis, Staphylococcus was significantly enriched in T cells and B cells (Figure S1F). Infected T cells exhibited increased expression of EEF1A2 (Figure S1G), a gene known to be hypomethylated in advanced GC^16^, with its overexpression associated with poor prognosis^17^. GO enrichment analysis indicated disruptions in autophagy and inflammatory processes (Figure S1H). GSEA revealed the activation of inflammasome-related pathways, including the interferon alpha (IFN-α) response and interferon gamma (IFN-γ) response (Figure S1I). Previous studies have shown that Helicobacter pylori infection induces upregulation of TLR9 and IFN-α expression in gastric epithelial cells and plasmacytoid dendritic cells^18^. Additionally, high levels of IFN-γ characteristic of CD8+ T cells can predict the response of GC patients to neoadjuvant immunotherapy combined with chemotherapy^19^.

In right-sided CRC tumor samples, Staphylococcus was predominantly identified in T cells, B cells, and neutrophils (Figure S1J). We observed an upregulation of CRTC1 and STAG3 expression in Staphylococcus-infected neutrophils (Figure S1k), aligning with previous reports that link CRTC1 activation to CRC progression^20^. Furthermore, STAG3 overexpression has been associated with poor prognosis, promoting metastasis and chemotherapeutic resistance in CRC.^21^. GO enrichment analysis revealed disruptions in phosphoserine residue binding and protein phosphorylated amino acid binding among these infected neutrophils (Figure S1L). GSEA results indicated that Staphylococcus-infected neutrophils activate inflammatory responses and immune regulatory processes (Figure S1M). These findings were consistent with earlier studies suggesting that a high-fat diet (HFD) rich in sunflower and coconut oils fostered a pro-inflammatory gut microenvironment, leading to an increased abundance of Staphylococcus and an elevated risk of CRC^22^.

In summary, these results suggested that Staphylococcus-specific infections in immune cells—such as T cells, B cells, and neutrophils—activate inflammatory processes and immune responses. This activation may play a role in the development of inflammation- and immunity-related diseases, including SLE, GC, and CRC.


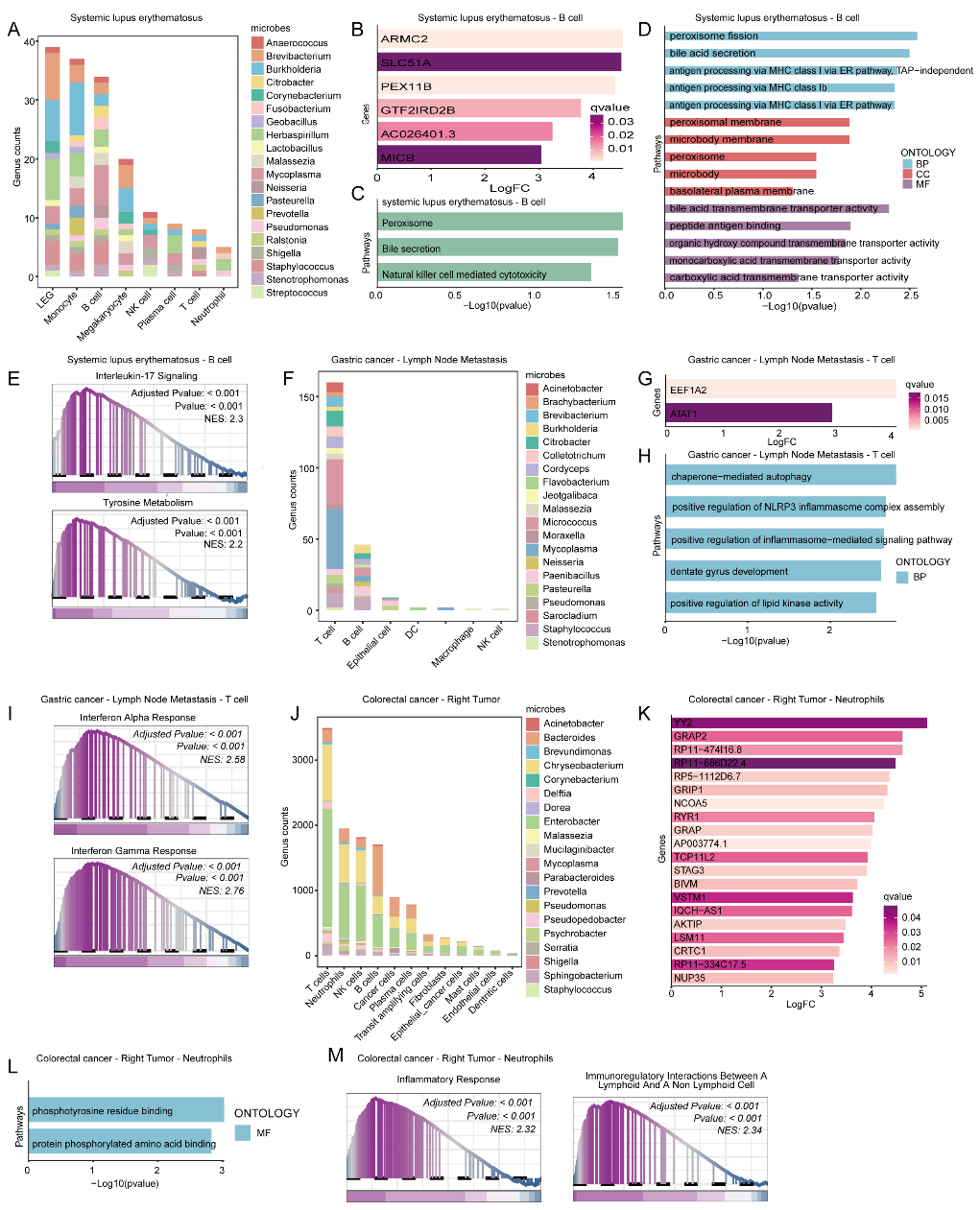


Figure S1. Utilize Staphylococcus as a case study to illustrate the utility and potential application in SLE, GC and CRC.

(A-E) Microbial distribution and biological processes associated with Staphylococcus infection in B cells of SLE. (A) Stacked bar plots illustrate the raw infection counts for each cell type; (B) Bar plot showing significantly DEGs in B cells with or without Staphylococcus infection; (C) Significantly enriched KEGG pathways for the DEGs from (B); (D) Significantly enriched GO pathways for the DEGs from (B); (E) GSEA plot highlighting the activation of interleukin-17 signaling and tyrosine metabolism pathways in B cells with or without Staphylococcus infection.

(F-I) Microbial distribution and biological processes associated with Staphylococcus infection in T cells of GC. (F) Stacked bar plots illustrate the raw infection counts for each cell type; (G) Bar plot showing significantly DEGs in T cells with or without Staphylococcus infection; (H) Significantly enriched GO pathways for the DEGs from (G); (I) GSEA plot highlighting the activation of interferon alpha response and interferon gamma response pathways in T cells with or without Staphylococcus infection. (J-M) Microbial distribution and biological processes associated with Staphylococcus infection in neutrophils of CRC. (J) Stacked bar plots illustrate the raw infection counts for each cell type. (K) Bar plot showing significantly DEGs in neutrophils with or without Staphylococcus infection; (L) Significantly enriched GO pathways for the DEGs from (K); (M) GSEA plot highlighting the activation of inflammatory response and immunoregulatory interactions between a lymphoid and a non-lymphoid cell pathways neutrophils.

**Future developments**

To better serve the broader biomedical research community, we plan several initiatives for the next version of ScMicrobesAtlas within the next two years:

**Collection and Integration of New single-cell sequencing data:** We aim to broaden our investigation of diseases by integrating additional single-cell sequencing data types to identify potential interactions and mechanisms associated with microbiome infections, particularly focusing on spatial transcriptome RNA sequencing (stRNA-seq). The incorporation of stRNA-seq data will allow us to explore the distribution and localized effects of microbiota within tumors and tissues, thereby enhancing our understanding of spatial, cellular, and molecular host-microbe interactions. By expanding our dataset to include diverse disease types and conditions, we seek to meet the research needs of a broader scientific community.

**Enhancement of Analytical Functionality：**We plan to enhance the existing analytical modules and develop advanced tools, including cell communication analysis, pseudotime analysis, and transcription factor analysis modules. These tools will allow us to investigate the effects of microorganisms on cell communication networks and their dynamic roles in cell development, differentiation, and disease progression. This will help elucidate the interactions between specific microbial infections and intracellular gene regulatory networks. Furthermore, the current version offers limited convenience for users comparing different datasets. To address this, we intend to introduce a more user-friendly "Compare" page to enhance the user experience.

Through these enhancements, we aim to improve the functionality and usability of ScMicrobesAtlas, ultimately facilitating a deeper understanding of the complex interactions between the microbiome and host cells across various disease contexts.

**Discussion**

Existing microbial databases, including GMrepo v2, gutMDisorder, GIMICA, DISBIOME, and curatedMetagenomicData, are crucial resources dedicated to the systematic collection and curation of disease-microbiome interactions. These databases aim to elucidate the complex mechanisms by which microbes influence human disease pathogenesis and progression. However, a notable limitation of these resources is their reliance on bulk tissue analyses, which obscures the intricate interactions between specific microbial communities and distinct host cell types. This approach has hindered a more granular understanding of the microbiome's role in shaping disease dynamics at the cellular level.

The effective analysis of microbe-host cell interactions at single-cell resolution provides invaluable insights into the mechanisms underlying disease pathogenesis. Ghaddar et al. utilized SAHMI to demonstrate that most microbes predominantly accumulate within tumor cells, significantly influencing host cell gene expression and activating tumor-specific immune responses^23^. Similarly, Galeano et al. employed in situ spatial detection techniques and scRNA-seq to investigate the spatial organization and cellular interactions of intratumoral microbial communities in oral squamous cell carcinoma and colorectal cancer. Their findings revealed that these highly structured microbial communities were preferentially localized within immunocompetent and epithelial cell-rich microenvironments, promoting cancer progression^24^.

ScMicrobesAtlas is a pioneering database that provides a comprehensive view of the microbiome in human diseases at single-cell resolution. This unique feature enables a comparative analysis of microbiome composition and facilitates the identification of both shared and cell-type-specific microbial enrichments across diverse cell types and disease states. Additionally, ScMicrobesAtlas empowers users to evaluate gene expression alterations and pathway activities within specific cell types in response to particular microbiomes, thereby enhancing the capacity to explore microbial-host interactions more effectively.”

**References**

1. Noguchi N, Ohashi T, Shiratori T, et al. Association of tannase-producing Staphylococcus lugdunensis with colon cancer and characterization of a novel tannase gene. *J Gastroenterol.* 2007;42(5):346-351.

2. Noguchi N, Fukuzawa M, Wajima T, et al. Specific clones of Staphylococcus lugdunensis may be associated with colon carcinoma. *J Infect Public Health.* 2018;11(1):39-42.

3. Wei Y, Sandhu E, Yang X, Yang J, Ren Y, Gao X. Bidirectional Functional Effects of Staphylococcus on Carcinogenesis. *Microorganisms.* 2022;10(12).

4. Shen Z, Dzink-Fox J, Feng Y, et al. Gastric Non-Helicobacter pylori Urease-Positive Staphylococcus epidermidis and Streptococcus salivarius Isolated from Humans Have Contrasting Effects on H. pylori-Associated Gastric Pathology and Host Immune Responses in a Murine Model of Gastric Cancer. *mSphere.* 2022;7(1):e0077221.

5. Yue K, Sheng D, Xue X, et al. Bidirectional Mediation Effects between Intratumoral Microbiome and Host DNA Methylation Changes Contribute to Stomach Adenocarcinoma. *Microbiol Spectr.* 2023;11(4):e0090423.

6. Mannion A, Sheh A, Shen Z, et al. Shotgun Metagenomics of Gastric Biopsies Reveals Compositional and Functional Microbiome Shifts in High- and Low-Gastric-Cancer-Risk Populations from Colombia, South America. *Gut Microbes.* 2023;15(1):2186677.

7. Hervier B, Ribon M, Tarantino N, et al. Increased Concentrations of Circulating Soluble MHC Class I-Related Chain A (sMICA) and sMICB and Modulation of Plasma Membrane MICA Expression: Potential Mechanisms and Correlation With Natural Killer Cell Activity in Systemic Lupus Erythematosus. *Front Immunol.* 2021;12:633658.

8. Morris DL, Taylor KE, Fernando MM, et al. Unraveling multiple MHC gene associations with systemic lupus erythematosus: model choice indicates a role for HLA alleles and non-HLA genes in Europeans. *Am J Hum Genet.* 2012;91(5):778-793.

9. Perl A. Oxidative stress in the pathology and treatment of systemic lupus erythematosus. *Nat Rev Rheumatol.* 2013;9(11):674-686.

10. Shah D, Sah S, Nath SK. Interaction between glutathione and apoptosis in systemic lupus erythematosus. *Autoimmun Rev.* 2013;12(7):741-751.

11. Teng X, Brown J, Morel L. Redox Homeostasis Involvement in the Pharmacological Effects of Metformin in Systemic Lupus Erythematosus. *Antioxid Redox Signal.* 2022;36(7-9):462-479.

12. Terui H, Yamasaki K, Wada-Irimada M, et al. Staphylococcus aureus skin colonization promotes SLE-like autoimmune inflammation via neutrophil activation and the IL-23/IL-17 axis. *Sci Immunol.* 2022;7(76):eabm9811.

13. Iwata S, Hajime Sumikawa M, Tanaka Y. B cell activation via immunometabolism in systemic lupus erythematosus. *Front Immunol.* 2023;14:1155421.

14. Ringheim GE, Wampole M, Oberoi K. Bruton's Tyrosine Kinase (BTK) Inhibitors and Autoimmune Diseases: Making Sense of BTK Inhibitor Specificity Profiles and Recent Clinical Trial Successes and Failures. *Front Immunol.* 2021;12:662223.

15. Shao WH, Cohen PL. The role of tyrosine kinases in systemic lupus erythematosus and their potential as therapeutic targets. *Expert Rev Clin Immunol.* 2014;10(5):573-582.

16. Shi Z, Guo X, Hu X, et al. DNA methylation profiling identifies epigenetic signatures of early gastric cancer. *Virchows Arch.* 2024;484(4):687-695.

17. Hassan MK, Kumar D, Naik M, Dixit M. The expression profile and prognostic significance of eukaryotic translation elongation factors in different cancers. *PLoS One.* 2018;13(1):e0191377.

18. Ding L, Chakrabarti J, Sheriff S, et al. Toll-like Receptor 9 Pathway Mediates Schlafen(+)-MDSC Polarization During Helicobacter-induced Gastric Metaplasias. *Gastroenterology.* 2022;163(2):411-425.e414.

19. Li S, Li K, Tian F, et al. A high interferon gamma signature of CD8(+) T cells predicts response to neoadjuvant immunotherapy plus chemotherapy in gastric cancer. *Front Immunol.* 2022;13:1056144.

20. Schumacher Y, Aparicio T, Ourabah S, et al. Dysregulated CRTC1 activity is a novel component of PGE2 signaling that contributes to colon cancer growth. *Oncogene.* 2016;35(20):2602-2614.

21. Sasaki M, Miyoshi N, Fujino S, et al. The meiosis-specific cohesin component stromal antigen 3 promotes cell migration and chemotherapeutic resistance in colorectal cancer. *Cancer Lett.* 2021;497:112-122.

22. Rodríguez-García C, Sánchez-Quesada C, Algarra I, Gaforio JJ. The High-Fat Diet Based on Extra-Virgin Olive Oil Causes Dysbiosis Linked to Colorectal Cancer Prevention. *Nutrients.* 2020;12(6).

23. Ghaddar B, Biswas A, Harris C, et al. Tumor microbiome links cellular programs and immunity in pancreatic cancer. *Cancer Cell.* 2022;40(10):1240-1253.e1245.

24. Galeano Niño JL, Wu H, LaCourse KD, et al. Effect of the intratumoral microbiota on spatial and cellular heterogeneity in cancer. *Nature.* 2022;611(7937):810-817.
